# Hyperpolarization of Long-Lived States of Protons in Aliphatic Chains by Bullet Dynamic Nuclear Polarization, Revealed on the Fly by Spin-Lock-Induced Crossing

**DOI:** 10.1021/acs.jpclett.4c01457

**Published:** 2024-08-27

**Authors:** Aiky Razanahoera, Anna Sonnefeld, Kirill Sheberstov, Pooja Narwal, Masoud Minaei, Karel Kouřil, Geoffrey Bodenhausen, Benno Meier

**Affiliations:** † Laboratoire des Biomolécules, LBM, Département de Chimie, École Normale Supérieure, PSL University, Sorbonne Université, CNRS, 75005 Paris, France; ‡ Institute of Biological Interfaces 4, Karlsruhe Institute of Technology, Eggenstein-Leopoldshafen 76344, Germany; § Institute of Physical Chemistry, Karlsruhe Institute of Technology, Karlsruhe 76131, Germany

## Abstract

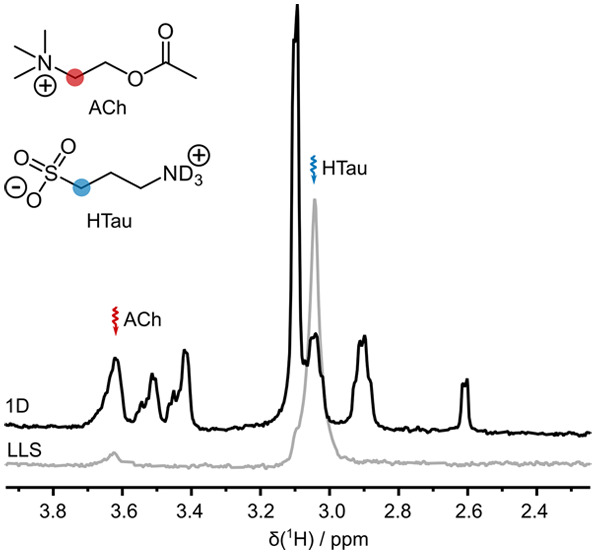

It is shown that proton spins highly polarized by dynamic nuclear polarization (DNP) retain substantial polarization upon the rapid transfer of frozen bullets from a polarizer to an NMR spectrometer. After injection in solution, the resulting hyperpolarization in aliphatic chains comprises population imbalances between singlet and triplet states of geminal protons and combinations thereof. These hyperpolarized long-lived states (LLSs) can be reconverted into observable transverse magnetization by polychromatic spin-lock-induced crossing (poly-SLIC). This reconversion can be achieved simultaneously in several molecules. Consecutive partial reconversion steps can be carried out to determine the lifetimes *T*_LLS_ on the fly in a single experiment. The enhancement factors of hyperpolarized LLS-derived signals in our experiments are at least 2 orders of magnitude. These methods extend applications of bullet-DNP to protons in molecules containing short aliphatic chains and may be useful for drug screening.

The development of methods to achieve nuclear spin hyperpolarization has greatly expanded applications of nuclear magnetic resonance (NMR).^1^ One of the most universal hyperpolarization approaches is known as dissolution dynamic nuclear polarization (d-DNP).^2^ In this technique, the electron polarization obtained at high fields and low temperatures is transferred to the nuclear spins of small molecules of interest in a frozen solid. This solid is then dissolved inside the polarizer, and the resulting solution is transferred to a liquid-state NMR spectrometer, where high-resolution NMR spectra are observed with signal intensities boosted by up to 4 orders of magnitude.^3^ Another implementation of DNP is the bullet-DNP (b-DNP) approach,^4,5^ where the sample is transferred to the liquid-state spectrometer as a frozen solid, followed by injection at high field. This implementation benefits from a fast sample transfer and improved scalability of the liquid sample volume to the volume of the used NMR detector.

Most frequently, both dissolution-DNP and bullet-DNP experiments are used to hyperpolarize heteronuclear spins such as ^13^C and ^15^N, which typically exhibit relaxation time constants *T*_1_ that are long enough in both liquids and solids to retain substantial hyperpolarization during sample transfer. For protons, dissolution-DNP has been mostly restricted to the observation of hyperpolarized fumarate^6^ and hyperpolarized HDO.^7^ The latter has a sufficient lifetime constant *T*_1_(^1^H) of ca. 15 s in solution while *T*_1_(^1^H) of H_2_O is only a few seconds. In the case of bullet-DNP, because the sample is transferred as a solid, proton relaxation is even more deleterious. This can be exacerbated at low magnetic fields and by high concentrations of radicals in the bullets. In the particular case of trityl, it was recently shown that the polarization of carbon-13 is largely retained during transfer, provided the bullet can be transferred in less than 100 ms.^4,5^ These findings are consistent with studies of the low-temperature, low-field relaxation behavior of samples containing trityl.^8,9^ No systematic studies of low-field, low-temperature relaxation of samples containing TEMPOL have been presented to date. One expects strong paramagnetic relaxation enhancement (PRE) to occur due to the high concentration of unpaired electrons in the sample, thus resulting in short nuclear relaxation times.

Problems resulting from short *T*_1_(^1^H) values may be resolved by exploiting spin states with prolonged lifetimes. In a pair of coupled spin-1/2 nuclei, a long-lived state (LLS)^10,11^ corresponds to a triplet–singlet imbalance (TSI) between populations of the singlet (|*S*_0_⟩ ≡ 1/√2 [|αβ⟩ – |βα⟩]) and the average population of the three triplet states (|*T*_+1_⟩ ≡ |αα⟩, |*T*_0_⟩ ≡ 1/√2 [|αβ⟩ + |βα⟩], and |*T*_–1_⟩ ≡ |ββ⟩). It has recently been discovered that LLSs can be excited in solution in a wide range of achiral or chiral molecules containing short aliphatic chains with magnetically inequivalent geminal pairs of protons.^12,13^ Note that in solution the PRE of these methylene protons caused by TEMPOL at concentrations below 6 mM does not shorten *T*_LLS_ below typical values of *T*_1_ measured in the absence of radicals.^14^ In aliphatic chains comprising several consecutive CH_2_ groups −(CH_2_)_*n*_–, long-lived population imbalances involving all 2*n* protons can be created by dissolving hyperpolarized solids in a high magnetic field as DNP can bring about a nonequilibrium distribution of the populations of the spin states of the ^1^H nuclei. In a somewhat simplified view, one could imagine that DNP causes each of the protons to be in the same pure state, m_*z*_ = +1/2 or −1/2, depending on the microwave irradiation frequency. For a pair of protons, the population of either |*T*_+1_⟩ or |*T*_–1_⟩ states can thus approach unity. After dissolution, the TSI will thus be hyperpolarized and may be as large as  in a 2-spin system.^15^

In dissolution-DNP, the sample is transferred between the polarizer and the NMR magnet as a liquid. Therefore, LLS can be used to preserve hyperpolarization during transport.^6,15,16^ If the singlet and triplet states are close to being eigenstates, there is no need for any external manipulation to sustain the LLS. In such cases, the lifetime of the LLS is likely to be favorable. Tayler et al.^15^ have shown how a long-lived triplet–singlet imbalance can be readily prepared at low spin temperatures in doubly ^13^C-labeled pyruvic acid, where the ^13^C spins are chemically inequivalent. Bornet et al.^6^ have exploited these properties for the two protons of fumarate that are inequivalent in the frozen lattice but turn into a magnetically equivalent pair upon dissolution. Dumez et al.^17^ used DNP to populate long-lived nuclear spin states in methyl groups in ^13^C-enriched pyruvate, which can be reconverted into observable signals by cross-relaxation involving both ^1^H and ^13^C. Related effects can be observed without DNP in molecules containing methyl groups with large tunneling splittings, such as acetate and gamma-picoline.^18−20^

By contrast, in bullet-DNP, the sample is transferred from the polarizer to the NMR spectrometer as a solid. Due to the lack of rapid molecular rotational diffusion and to the presence of inter- and intramolecular dipolar couplings, neither singlet nor triplet states are eigenstates in the solid, so that one cannot refer to singlet–triplet imbalances. It is only after the rapid dissolution of the bullet in a warm solvent that the non-Boltzmann distribution of populations may comprise singlet–triplet imbalances that can have long lifetimes.

While previous bullet-DNP experiments have been limited to the transfer of hyperpolarized low-γ nuclei such as carbon-13 in the presence of the narrow-band radical OX063, in this work, we show that (i) highly polarized *proton* spins can retain a substantial fraction of their polarization upon transfer of frozen bullets from the polarizer to the NMR spectrometer despite the presence of the wide-band radical TEMPOL. We furthermore show that (ii) a solvent volume of 350 μL (as opposed to 5 mL typically used for dissolution DNP) is sufficient to dissolve a hyperpolarized solid; (iii) long-lived population imbalances comprised in the hyperpolarized state are conserved; (iv) these LLSs can be reconverted into observable magnetization by polychromatic spin-lock-induced crossing (poly-SLIC);^13^ (v) this can be achieved in several molecules simultaneously; and (vi) the reconversion can be achieved in several consecutive fractions to allow an on-the-fly determination of lifetimes *T*_LLS_ in a single experiment.

In the pulse sequence shown in Figure 1, an initial ^1^H free induction decay (FID), excited by a small flip angle pulse β, is recorded 1.4 s after the arrival of the hyperpolarized sample while turbulences are settling down, allowing an estimation of the level of proton hyperpolarization without affecting the LLS. A *T*_00_ filter^21^ is applied every 1.2 s, which roughly matches *T*_1_(^1^H) of the observed molecules, in order to minimize contributions of magnetization to LLS-derived signals. These filters consist of hard pulses interleaved with gradients and efficiently destroy unwanted signals. To detect the decay of LLS on the fly, one can use cascades of short SLIC pulses. By reducing the duration of the RF irradiation from the optimum (e.g., from τ_SLIC_^opt^ = 110 ms to τ_SLIC_^θ^ = 10 ms for DSS), one can reduce the fraction of the long-lived states that is reconverted to observable magnetization. This is similar to the reduction of the flip angle of a conventional excitation pulse that can be used for on-the-fly inversion–recovery measurements. We refer to such cascades as SLIC(θ). This opens the way to on-the-fly observation of the decay of long-lived states and hence to the determination of their lifetimes *T*_LLS_ using a single hyperpolarized sample.

**Figure 1:**
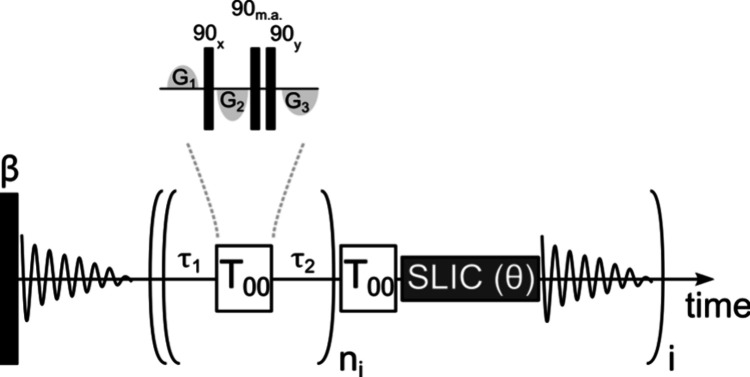
Pulse sequence appropriate for on-the-fly detection of the decay of LLS by multiple short SLIC(θ) pulses. Immediately after receiving a trigger signal from the bullet control system, an excitation pulse with a small flip angle β is applied to measure the ^1^H polarization without saturating the receiver. A series of LLS-derived spectra are then acquired by applying a cascade of SLIC(θ) pulses. A cascade of *T*_00_ filters^21^, each filter lasting 9.5 ms, is inserted before each SLIC(θ) pulse in order to minimize contributions to the signals that stem from magnetization rather than from LLS. Each *T*_00_ filter is sandwiched between two delays τ_1_ and τ_2_, which are chosen depending on *T*_1_, thus forming a block that is repeated *n*_*i*_ times. The value of *n*_*i*_ can be increased as the intervals between the SLIC(θ) pulses are increased. The procedure is repeated *i* = 1···*m* times to determine the decay of the LLS-derived signals. In each *T*_00_ filter, the durations and amplitudes of the three gradients are 4.4 ms, 2.4 ms, 2 ms and 0.56 G/cm, −1.1 G/cm, −0.84 G/cm.

Two bullet samples containing acetylcholine (AcCh), ethanolamine, and trimethylsilylpropanesulfonic acid (DSS) were dissolved in DNP juice. In separate experiments, these samples were hyperpolarized and shot as solids into an NMR tube containing methanol-*d*_4_ in a liquid-state spectrometer (samples I and II).

The enhancement factors for the magnetization were determined by comparing integrals of peaks in the hyperpolarized spectrum with a reference spectrum acquired under the same conditions after the complete return to thermal polarization (i.e., after the complete decay of the hyperpolarization). Under thermal mixing conditions in the DNP polarizer, all spins should reach the same spin temperature (i.e., the same level of polarization that is proportional to the inverse spin temperature). Once the sample is dissolved, however, the relaxation rates can be different for different spins, causing the remaining enhancement factor to be larger for spins with longer *T*_1_. The enhancement factors were determined for the methyl peak of DSS, which has *T*_1_ = 2.0 s, the longest among the solute signals. In solution, the enhancement factors of the magnetization were ε^I^ = 336 for sample I and ε^II^ = 269 for sample II at 298 K in a field of 9.4 T (400 MHz for ^1^H). This corresponds to proton polarization levels of *p*^I^ = 1.1% and *p*^II^ = 0.9% for samples I and II, respectively. Figure 2 shows a comparison of a thermal spectrum with a bullet-DNP enhanced spectrum of sample II.

**Figure 2:**
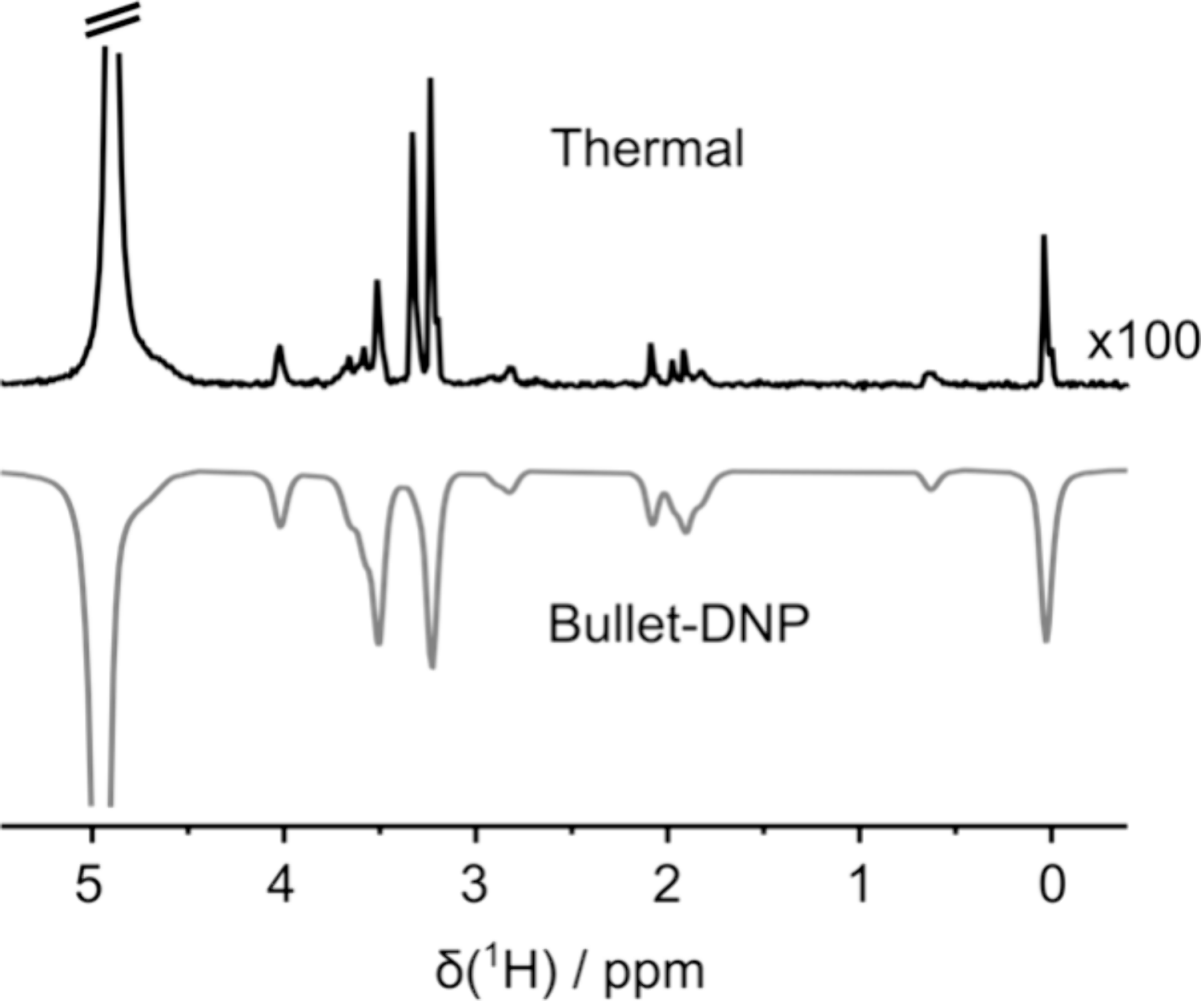
Thermal equilibrium spectrum of sample II with the vertical scale amplified 100-fold (top), compared to the hyperpolarized spectrum of the same sample 1.4 s after sample ejection from the polarizer (bottom). The hyperpolarized signals are negative since microwave irradiation was applied at the negative lobe of the DNP spectrum of TEMPOL.

Figure 3 (a) and (c) show spectra obtained by simultaneously monitoring the decay of LLS in the two molecules AcCh and DSS in samples I and II using SLIC(θ) sequences.

**Figure 3:**
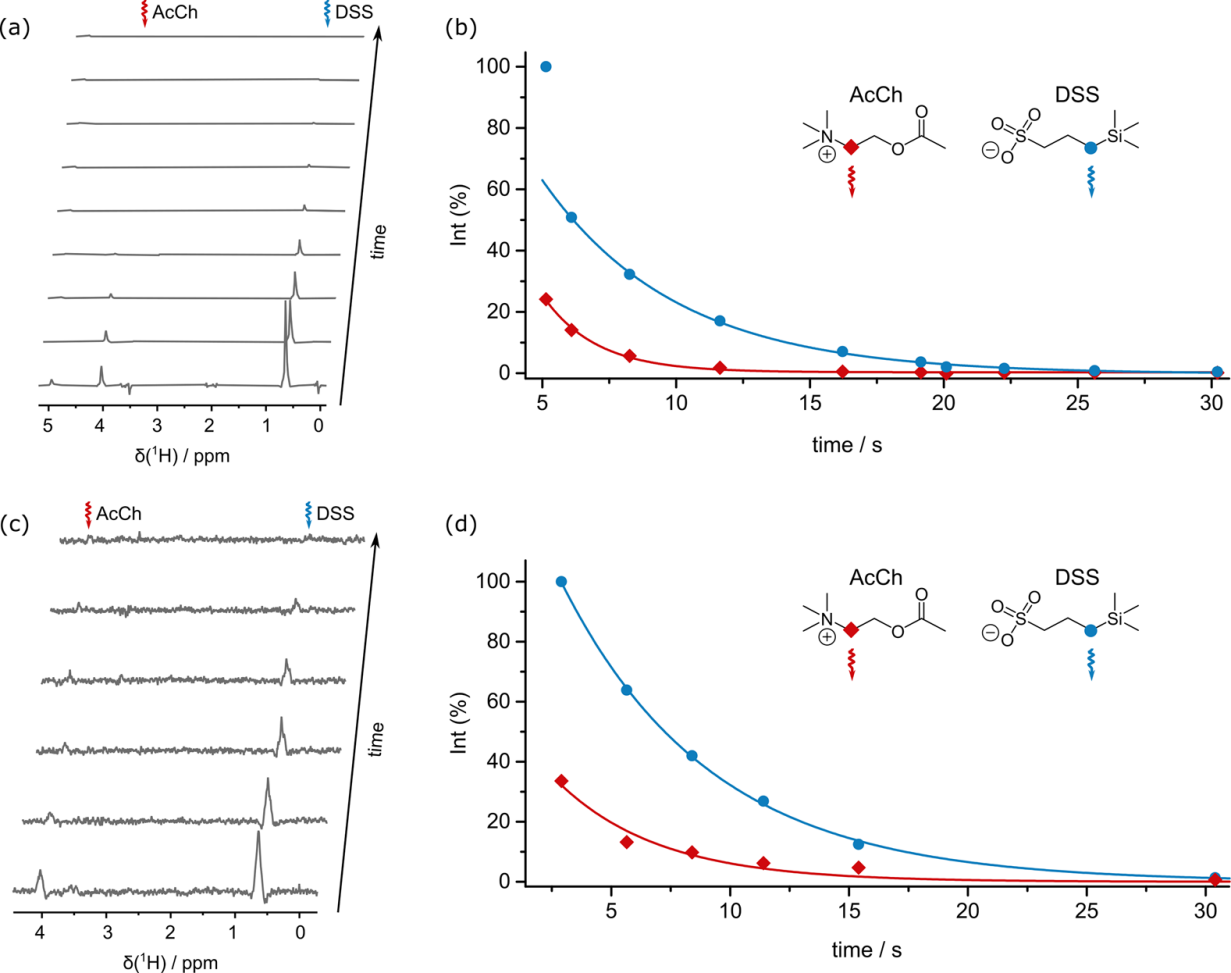
Spectra recorded using a cascade of SLIC(θ) pulses to reconvert fractions of LLS into transverse magnetization to monitor the decay of the LLSs on the fly, simultaneously in acetylcholine (AcCh) and DSS contained in (a) sample I using τ_SLIC_^θ^ = 50 ms and (c) sample II using τ_SLIC_^θ^ = 10 ms. The pulse sequence used is equivalent to the one shown in Figure 1, but the delays between acquisitions have been encoded manually, with a *T*_00_ filter being applied at least every 1.2 s and immediately before each acquisition. The irradiated multiplets are indicated by wavy arrows, pointing to the targeted CH_2_ groups in the molecular structures. The integrals of the LLS-derived signals were plotted as a function of time after injection for samples I (b) and II (d) and fitted to a monoexponential function (for DSS in (b), where the first data point was excluded from the fit, because of a discrepancy attributed to residual convection). The determined lifetimes were *T*_LLS_ = 2.0 ± 0.1 s in AcCh and *T*_LLS_ = 5.0 ± 0.2 s in DSS for sample I and *T*_LLS_ = 4.3 ± 0.7 s in AcCh and *T*_LLS_ = 6.3 ± 0.2 s in DSS for sample II. The line widths (full widths at half-maxima) of the LLS-derived signals for DSS were ca. 12 Hz for sample I (first spectrum in (a)) and ca. 40 Hz for sample II (first spectrum in (c)).

The integrals of the LLS-derived signals were plotted as a function of time and fitted to monoexponential functions (Figure 3 (b) and (d)). The apparent lifetimes were determined to be *T*_LLS_ = 2.0 ± 0.1 s for AcCh and *T*_LLS_ = 5.0 ± 0.2 s for DSS for sample I when using τ_SLIC_^θ^ = 50 ms. The *T*_LLS_ values were found to be slightly longer if τ_SLIC_^θ^ = 10 ms in sample II. The determined time constants were *T*_LLS_ = 4.3 ± 0.7 s for AcCh and *T*_LLS_ = 6.3 ± 0.2 s for DSS. This is in agreement with the expectation that the partial conversion of long-lived order into magnetization in each SLIC(θ) block shortens the apparent *T*_LLS_. Also of note is the fact that the intensities of the LLS-derived signals of AcCh were much lower than those of DSS in both experiments, even though the former was present at a 2-fold higher concentration. This is attributed to its shorter *T*_LLS_ and possibly to faster relaxation in the solid state during the bullet transfer. In both samples, the enhancements of the LLS-derived signals were determined by comparison with the corresponding LLS-derived signals of the thermally polarized samples after the decay of the hyperpolarization. These enhancement factors were above 100 for both molecules and both samples. Such enhancements allow the acquisition of on-the-fly measurements using SLIC, which would otherwise not be feasible in a timely manner under thermally polarized conditions even at molar concentrations.

Since the lifetime *T*_LLS_ is sensitive to binding events,^22−25^ a possible application for on-the-fly monitoring is the detection of protein–ligand binding using a hyperpolarized ligand.^24,26^ For such experiments, the used solvent must be compatible with the protein system, in most cases water or a physiological buffer solution.

A sample containing AcCh and homotaurine (HTau) was hyperpolarized and shot as a solid into a reservoir containing D_2_O, and the resulting solution was injected into an NMR tube. Figure 4 shows the LLS-derived signals of HTau and AcCh (gray spectrum) for this solution recorded with τ_SLIC_ = 150 ms and ν_SLIC_ = 55 Hz at 2.6 s after the ejection of the hyperpolarized solid from the polarizer. In this case, the duration and amplitude of the SLIC pulse correspond to average and sum, respectively, of the optimal parameters for HTau (τ_SLIC_^opt^ = 180 ms and *v*_SLIC_^opt^ = 26 Hz) and AcCh (τ_SLIC_^opt^ = 120 ms and *v*_SLIC_^opt^ = 29 Hz). The average SLIC parameters were used to reconvert the largest possible amount of LLS of both molecules using double SLIC. The LLS-derived signals are compared to a thermal spectrum recorded after the complete decay of the hyperpolarization (Figure 4, black spectrum).

**Figure 4:**
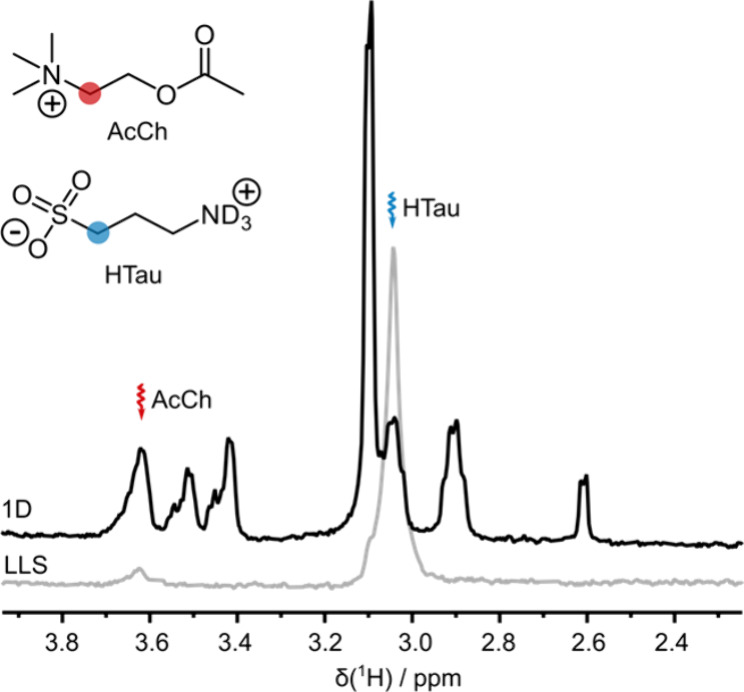
Thermal spectrum (black) and hyperpolarized spectrum resulting from the reconversion of LLS (gray) acquired at τ_0_ = 2.6 s after bullet ejection. A single poly-SLIC pulse with τ_SLIC_ = 150 ms and ν_SLIC_ = 55 Hz was applied at the offsets indicated by the wavy arrows.

Note that the intensity of the LLS-derived signal of HTau in this experiment was greater than that of the corresponding signal at thermal equilibrium. Without hyperpolarization, LLS-derived signals of aliphatic 4- or 6-spin systems typically reach less than 10% of the thermal signal intensity.

This work shows that proton spin hyperpolarization boosted by DNP is substantially retained during the rapid transfer of frozen solid bullets. The resulting hyperpolarization in solution comprises population imbalances between the singlet and triplet states of geminal protons in aliphatic chains. The boosted LLS may be reconverted in small fractions into observable magnetization to determine the lifetimes *T*_LLS_ on the fly. In the presence of fast chemical exchange between a free and a protein-bound form, the *T*_LLS_ of the ligand may drop dramatically, as the spin symmetry changes upon complexation, thus providing a high contrast upon binding.^22−25^ On-the-fly determination of *T*_LLS_ time constants of hyperpolarized LLSs in the presence of variable concentrations of a ligand can therefore be useful for drug screening.

## Methods

**Figure 5:**
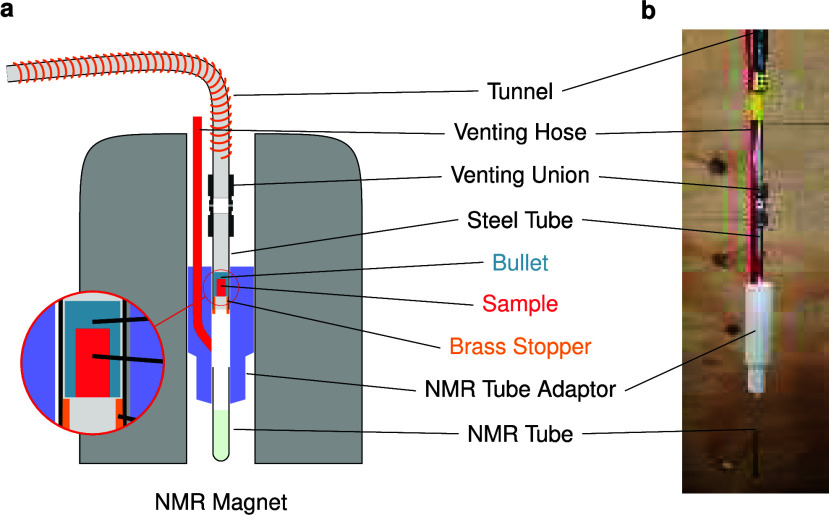
Sketch (a) and photograph (b) of the injection device used in this work. The sample is placed into a small Teflon bucket (the “bullet”, light blue), and polarized using the instrumentation (not shown) described in ref (5). The NMR tube is prefilled with solvent and fixed inside the 3D-printed NMR tube adaptor using Teflon tape. After polarization has built up sufficiently, the tunnel between the polarizer and the injection device is energized to provide a field of approximately 60 mT, and the sample is shot through the tunnel into the injection device within 70 ms by using pressurized helium. Small holes in the venting union allow helium gas to escape. A steel tube is connected to the bottom of the venting union. A small brass stopper with a constricted diameter is brazed to the bottom of this steel tube. As visible in the magnified view on the bottom left, the bullet itself cannot pass the brass stopper, but the “naked” sample (red) travels by inertia through the constriction, is fragmented upon impact on the liquid surface, and dissolves upon its immersion in the ambient-temperature solvent that is contained in the NMR tube. The NMR acquisition is started automatically 1.4 s after the ejection of the sample from the polarizer.

Hyperpolarization was performed at a sample temperature of 1.55 K in a field of 6.7 T with microwave saturation above or below the maximum in the EPR spectrum of the stable radical TEMPOL. When the sample is shot into D_2_O, a standard injection device with a reservoir is used. When the sample is shot into MeOD, another injection device is used, as shown in Figure 5. In order to start signal acquisition as soon as possible, we omit the use of a reservoir^5^ except for measurements where dissolution occurred in water. After the ejection of the 50 μL hyperpolarized sample from the bullet casing, it travels directly into a 5 mm OD NMR tube, which is preloaded with 350 μL of methanol-*d*_4_ (CD_3_OD), thus achieving a 2-fold reduction in solvent volume (hence avoiding unnecessary dilution) compared to earlier studies.

For the preparation of samples I and II, the bullets were loaded with 50 μL of a mixture of 195 mM (14.1 mg) acetylcholine (AcCh), 200 mM (4.8 μL) ethanolamine, and 95 mM (8.3 mg) trimethylsilylpropanesulfonic acid (DSS) in a “DNP juice” containing 40 mM TEMPOL, D_2_O, H_2_O, and glycerol-*d*_8_ in a volume ratio of 30/10/60. The frozen samples had a glassy aspect, indicating that they were not crystalline, which is essential for efficient spin diffusion in the proton bath. After hyperpolarization, the bullets were shot directly into 350 μL of methanol-*d*_4_ at room temperature. In one case, the transfer of the DNP juice from the bullet to the NMR tube was incomplete, as *ca*. 10 μL of the 50 μL sample remained behind in the bullet because of incomplete ejection. Therefore, the concentrations in methanol solution were approximately 20 mM AcCh, 9.7 mM DSS, 21 mM ethanolamine, and 4.1 mM TEMPOL for sample I and 24 mM AcCh, 12 mM DSS, 25 mM ethanolamine, and 5.0 mM TEMPOL for sample II.

For applications to drug screening, dissolution in water (or in a physiological buffer solution) is required to maintain the protein integrity. Since water has a tendency to trap air bubbles, the injection device described in ref (5) was used for experiments with D_2_O. In these experiments, the frozen sample containing 231 mM (16.8 mg) AcCh and 293 mM (16.3 mg) HTau in the same DNP juice was shot into a reservoir filled with 700 μL of D_2_O, which was positioned approximately 20 cm above the magnetic center of the magnet where the field strength is nearly 1 T. Approximately 1.5 s after the arrival of the bullet, 300 μL of the liquid was injected into a 5 mm NMR tube, and the NMR acquisition was triggered 2.6 s after ejection of the hyperpolarized solid from the polarizer. The resulting solution contained *ca*. 15 mM AcCh, 20 mM HTau, and 3 mM TEMPOL. The field-frequency lock was switched off for LLS measurements in D_2_O.

## Data Availability

Research data shown in this manuscript are available at KITopen (DOI 10.35097/6h9v9z84stf9633v).

## Supporting Information Available

Transparent Peer Review report available (PDF)
