# Supplementary material for: Hyperpolarization of Long-Lived States of Protons in Aliphatic Chains by Bullet Dynamic Nuclear Polarization, Revealed on the Fly by Spin-Lock-Induced Crossing
Source: J Phys Chem Lett. 2024 Aug 27;15(35):9024–9. doi: 10.1021/acs.jpclett.4c01457 (PMC11626513; doi:10.1021/acs.jpclett.4c01457)
Supplement: Supplementary file 1 — jz4c01457_si_001.pdf [file jz4c01457_si_001.pdf]

jz-2024-014578.R1

Name: Peer Review Information for "Hyperpolarization of Long-Lived States of Protons in Aliphatic Chains by Bullet Dynamic Nuclear Polarization, Revealed On-The-Fly by Spin-Lock Induced Crossing"

First Round of Reviewer Comments

Reviewer: 1

Comments to the Author

Comments on jz-2024-014578

The authors show that triplet-singlet population imbalances of geminal proton pairs in aliphatic chains are protected from relaxation in the solid state, and are thus able to survive transfer between a DNP polarizer and an NMR spectrometer via bullet DNP. They also demonstrate on-the-fly measurement of LLS relaxation after dissolution via a train of partial SLIC pulses, much in the same way that hyperpolarized magnetization is read out via a series of small-angle pulses. The authors also report a technical improvement in their instrumentation that allows for dissolution into a smaller volume of solvent than used previously.

The main result, that triplet-singlet imbalances in aliphatic chains appear to be long-lived in the solid state during bullet transport, is interesting, suggesting that what is a LLS in solution may also be protected from paramagnetic relaxation even in the absence of molecular tumbling. Unfortunately, the authors don't offer much analysis of how or why this is the case. While I am very much in favor of short and sweet manuscripts, this one really left me wanting more. And in order to justify publication in *J. Phys. Chem. Lett.*, I feel like the authors really need to dig into the physics of what's going on. Perhaps varying the guiding field in the solenoid wrapped around the shuttling tube could provide some information, as different values of the Zeeman energy vs. the dipole-dipole energy could affect relaxation rates in an informative way? Or changing the transfer time (shooting the bullet with less pressure?) could provide some insight into how the polarization is relaxing?

If the authors prefer to leave the manuscript as "we observed this interesting result" without further investigation, a more specialized journal might be more appropriate. That said, the current work is technically sound and clearly written, so I have no opposition to publication following some minor revisions.

Additional comments and suggestions are given below.

Additional Comments:

1. The SNR in Fig. 3(c) is surprisingly low, which is confusing given the high SNR of the spectrum of thesame sample in Fig. 2. The main difference seems to be that $\tau_{SLIC}{}^{\theta}$ = 10ms instead of 50ms. It seems like the effective flip angle is not linear inτ_{SLIC}^{θ} – the authors should explain what the effective flip angles are, and perhaps how to calculate them.
2. It would be helpful if the authors could state what the initial polarization of the singlet-triplet imbalance wasin the DNP polarizer ($-1/3$?) compared to what remained upon arrival in the liquid-state spectrometer. My best guess is that the polarization decreased by a factor of about 30x. What caused this loss, and what could be done to preserve more of the initial polarization?

3. It is unclear to me whether bullet DNP offers an advantage over dissolution DNP for preserving LLS inthese molecules – have similar measurements been performed, and how do the different techniques compare (i.e. fraction of polarization remaining upon arrival in the liquid-state spectrometer)? If the manuscript is meant to be just about the physics of the process, it doesn't really matter if the technique is better than a given alternative, but it would still be useful to comment.

Minor suggestions:

1. In the introduction, line 45/46, "achiral or chiral" seems like a somewhat unnecessary modifier to the word "molecules".

Author's Response to Peer Review Comments:

Laboratoire des Biomolécules – LBM (UMR 7203)
Département de Chimie
Ecole Normale Supérieure
Université PSL
24 Rue Lhomond
75005 Paris CEDEX 05
kirill.sheberstov@ens.psl.eu
Geoffrey.bodenhausen@ens.fr

Paris, June 25th 2024

Re : Journal: The Journal of Physical Chemistry Letters
Manuscript ID: jz-2024-014578
Original Submission Date: 17-May-2024
Title: "Hyperpolarization of Long-Lived States of Protons in Aliphatic Chains by Bullet Dynamic Nuclear Polarization, Revealed On-The-Fly by Spin-Lock Induced Crossing"
Author(s): Razanahoera, Aiky; Sonnefeld, Anna; Sheberstov, Kirill; Narwal, Pooja; Minaei, Masoud; Kouril, Karel; Bodenhausen, Geoffrey; Meier, Benno

Dear Editor

Please find below our point-by-point responses to the reviewer's comments. Your letter is reproduced in black, our responses in blue:

Thank you for submitting your manuscript for publication in The Journal of Physical Chemistry Letters. It has been examined by an expert reviewer who concluded that the work is of potential interest to the readership of The Journal of Physical Chemistry Letters; however, it appears that a major revision, possibly followed by further reviewer evaluation, will be needed prior to its further consideration for publication. Please see the enclosed reviewer's report for details regarding the requested changes and/or additions.

Reviewer(s)' Comments to Author:

Reviewer: 1

Recommendation: While the work is good and publishable, a more appropriate journal is recommended such as J. Magn. Reson.

We believe that the significance of our observations warrants publication for a wider audience.

Comments:
See attached

Additional Questions:
Urgency: High

Significance: High

Novelty: High

Scholarly Presentation: High

Is the paper likely to interest a substantial number of physical chemists, not just specialists working in the authors' area of research?: Yes

----
Comments on jz-2024-014578
The authors show that triplet-singlet population imbalances of geminal proton pairs in aliphatic chains are protected from relaxation in the solid state, and are thus able to survive transfer between a DNP polarizer and an NMR spectrometer via bullet DNP. They also demonstrate on-the-fly measurement of LLS relaxation after dissolution via a train of partial SLIC pulses, much in the same way that hyperpolarized magnetization is read out via a series of small-angle pulses.

The reviewer's comments reveal that he misunderstood some of the reasoning in our paper. In the solid state, the molecules cannot tumble freely, and they cannot freely explore the space of accessible conformations. As a result, the triplet and singlet states are not eigenstates in the solid state. We cannot say that triplet-singlet population imbalances are long-lived in the solid state, and do not argue that these population imbalances are the mechanism by which we obtain signal after the transfer between the DNP polarizer and the NMR spectrometer.

Since the reviewer appears to be well informed on matters of hyperpolarization, we conclude that our text was not sufficiently clear. We have therefore inserted a paragraph on p. 4 to explain this in more detail: "*By contrast, in bullet-DNP, the sample is transferred as a solid. Due to the lack of rapid molecular rotational diffusion and to the presence of inter- and intra-molecular dipolar couplings, neither singlet nor triplet states are eigenstates in the solid, so that it is not useful to refer to singlet-triplet imbalances. It is only after the transfer of the bullet from the polarizer to the NMR spectrometer and its rapid dissolution in a warm solvent that the non-Boltzmann distribution of populations comprises singlet-triplet imbalances that can have long lifetimes.*"

The authors also report a technical improvement in their instrumentation that allows for dissolution into a smaller volume of solvent than used previously.

Indeed, our paper describes a number of technical improvements of our instrumentation.

The main result, that triplet-singlet imbalances in aliphatic chains appear to be long-lived in the solid state during bullet transport, is interesting, suggesting that what is a LLS in solution may also be protected from paramagnetic relaxation even in the absence of molecular tumbling.
Unfortunately, this interpretation is too optimistic. In fact, the hyperpolarization is *NOT* protected from paramagnetic relaxation in the solid phase. However, our study does indeed show that hyperpolarized proton spin polarization can be transferred and subsequently observed in the presence of paramagnetic TEMPOL. This finding alone substantially widens the applicability of hyperpolarization techniques. We now explain this finding more explicitly on p. 4:
"*While previous bullet-DNP experiments have been limited to the transfer of hyperpolarized low-γ nuclei like carbon in the presence of the narrow-band radical OX063, in this work, we show that (i) highly polarized proton spins can retain a substantial fraction of their polarization upon transfer of frozen bullets from the polarizer to the NMR spectrometer in the presence of the wide-band radical TEMPOL.*"

Unfortunately, the authors don't offer much analysis of how or why this is the case. While I am very much in favor of short and sweet manuscripts, this one really left me wanting more. And in order to justify publication in J. Phys. Chem. Lett., I feel like the authors really need to dig into the physics of what's going on. Perhaps varying the guiding field in the solenoid wrapped around the shuttling tube could provide some information, as different values of the Zeeman energy vs. the dipole-dipole energy could affect relaxation rates in an informative way? Or changing the transfer time (shooting the bullet with less pressure?) could provide some insight into how the polarization is relaxing?
A satisfactory analysis of relaxation in the solid state would be very demanding. Because of the presence of TEMPOL radicals, paramagnetic relaxation is likely to be predominant. The 'bullet' samples are ejected from the polarizer and shot through a magnetic tunnel to the NMR spectrometer where the solid sample is dissolved. During the 'voyage', the temperature increases from ca 1.4 K to 20-40 K, while the sample is exposed to time-dependent magnetic fields (the magnetic tunnel cannot guarantee a constant field.) Finally, the sample is dropped into a solvent at room temperature where it is supposed to disintegrate abruptly. It is likely that smaller fragments of the frozen sample dissolve more rapidly than large chunks, so that the dissolution process is not instantaneous. Only *after* dissolution can molecular tumbling lead to averaging of the intramolecular dipole-dipole interactions and of the chemical shift anisotropy (CSA) of the protons, while internal rotations about carbon-carbon bonds can lead to partial averaging of J-couplings and hence to the appearance of AA'MM'XX' systems in aliphatic chains.

If the authors prefer to leave the manuscript as "we observed this interesting result" without further investigation, a more specialized journal might be more appropriate.
Indeed, we primarily wish to report that "we observed this interesting result" without further investigation, not by lack of curiosity about the mechanisms, but because of their extreme complexity (*vide supra*).

That said, the current work is technically sound and clearly written, so I have no opposition to publication following some minor revisions.
We hope that the Editor will accept that our proposed revisions are not as ambitious as the reviewer wishes.

Additional comments and suggestions are given below.

Additional Comments:
1. The SNR in Fig. 3(c) is surprisingly low, which is confusing given the high SNR of the spectrum of the same sample in Fig. 2. The main difference seems to be that τ_{SLIC} θ = 10 ms instead of 50 ms. It seems like the effective flip angle is not linear in τ_{SLIC} θ – the authors should explain what the effective flip angles are, and perhaps how to calculate them.
The difference in SNR is due to the combined effect of the shorter τ_{SLIC} and a superior shim / linewidth in experiment (a). An addition to the caption of Figure 3 now points out the difference in linewidths:
"The linewidths (full width at half maximum) of the LLS-derived signals for DSS were ca. 12 Hz for sample I (first spectrum in (a)) and ca. 40 Hz for sample II (first spectrum in (c))."

2. It would be helpful if the authors could state what the initial polarization of the singlet-triplet imbalance was in the DNP polarizer (−1/3?) compared to what remained upon arrival in the liquid-state spectrometer.
It is difficult to estimate the initial (Zeeman) polarization. The partial conversion of this polarization into a singlet-triplet imbalance constitutes a proof that the populations do not obey a linear function of the Zeeman energy, i.e., that the distribution of populations violates the high temperature approximation.

My best guess is that the polarization decreased by a factor of about 30x. What caused this loss, and what could be done to preserve more of the initial polarization?
The losses are due mostly to paramagnetic relaxation in the solid bullet as it travels through low magnetic fields while it warms up.

3. It is unclear to me whether bullet DNP offers an advantage over dissolution DNP for preserving LLS in these molecules – have similar measurements been performed, and how do the different techniques compare (i.e. fraction of polarization remaining upon arrival in the liquid-state spectrometer)?
We have tried to compare dissolution DNP with bullet DNP. In the former case, the sample is dissolved in the polarizer and travels through the magnetic tunnel as a liquid. A partial conversion of the non-Boltzmann distribution of populations occurs spontaneously at the time of dissolution. The conversion of hyperpolarized Zeeman order into long-lived states (LLS) can also be brought about in the NMR spectrometer by selective irradiation. At this time, the two approaches appear to provide similar enhancement, but less material is needed for bullet DNP experiments due to the lower solvent volume.

If the manuscript is meant to be just about the physics of the process, it doesn't really matter if the technique is better than a given alternative, but it would still be useful to comment.
The mere finding that dissolution DNP and bullet DNP can deliver similar results is likely to stimulate further developments.

Minor suggestions:
1. In the introduction, line 45/46, "achiral or chiral" seems like a somewhat unnecessary modifier to the word "molecules".
We would like to keep these apparently superfluous adjectives because it is remarkable that SLIC works both in chiral molecules (as shown by DeVience et al.) as well as in achiral molecules, as we have shown ourselves.

Please also make the following non-scientific changes:
1) TOC Graphic: Provide a TOC image per journal guidelines (2 in x 2 in; on the same page as the abstract) with the heading "TOC Graphic" above the graphic. The graphic should be in the form of a structure, graph, drawing, photograph, or scheme—or a combination. Non-scientific cartoon-like images or caricatures are discouraged. https://pubsapp.acs.org/paragonplus/submission/toc_abstract_graphics_guidelines.pdf
We have inserted a TOC figure just after the abstract, along with a brief caption.

2) References: In both the main file and the supporting information, fix the style of all references to use JPCL formatting (check all references carefully). ***JPC Letters reference formatting requires that journal references should contain: () around numbers; author names; article title (titles entirely in title case or entirely in lower case); abbreviated journal title (italicized); year (bolded); volume (italicized); and pages (first-last). Book references should contain author names; book title (in the same pattern); publisher; city; and year. Websites must include date of access.
We have cited all article titles in title case.

3) Please submit your publication files without any markups. Please include annotated version(s) of your revised publication file(s) with colored text or highlights indicating the revisions that you have made as "Supporting Information for Review Only."
We submit (1) a DOC file without any markups, and (2) a DOC file where all changes have been highlighted.

When submitting your revised manuscript through ACS Paragon Plus, you will be able to respond to the reviewer and editorial comments in the text box provided or by attaching a file containing your detailed responses outlining the changes made and explaining your reasons for disagreeing with any suggestions you choose not to follow.

We allow 30 days for revision, but we encourage you to submit within two weeks. If further delays are necessary, please notify us as soon as possible. Here at JPC Letters we try to expedite the processing of your manuscript, so your prompt response is greatly appreciated.

During submission, you were given a choice to participate in transparent peer review. You responded as follows:

Yes, I will participate in transparent peer review.
We do not wish to change our mind.

Supporting Information: If the manuscript is accompanied by any supporting information for publication, a brief description of the supplementary material is required in the manuscript. The appropriate format is: Supporting Information. Brief statement in nonsentence format listing the contents of the material supplied as Supporting Information.
There is no Supporting Information associated with this paper.

Funding Sources: Authors are required to report ALL funding sources and grant/award numbers relevant to this manuscript.
All funding sources have been indicated.

ORCID: Authors submitting manuscript revisions are required to provide their own validated ORCID iDs before completing the submission,
All authors have indicated their ORCID numbers.

We also require a graphic for the "Table of Contents" with all submissions. We ask that you include a graphic immediately after the Abstract under the header “TOC Graphic.” Instructions are available at https://publish.acs.org/publish/author_guidelines?coden=jpclcd .
We have inserted a TOC figure just after the abstract, along with a brief caption

We have made a few minor linguistic corrections. We hope that you will find the revised manuscript suitable for publication.

Sincerely,

Dr Benno Meier
Institute for Biological Interfaces 4
Karlsruhe Institute of Technology

Dr Kirill Sheberstov
Chargé de Recherche @ CNRS
Laboratoire des BioMolécules – LBM (UMR 7203)
Département de Chimie
Ecole Normale Supérieure
Université PSL
24 Rue Lhomond
75005 Paris

Prof. Dr Emeritus Geoffrey Bodenhausen
Laboratoire des BioMolécules – LBM (UMR 7203)
Département de Chimie
Ecole Normale Supérieure
Université PSL
24 Rue Lhomond
75005 Paris
